# Patient Perceptions of a Group-Based Lifestyle Intervention for Overweight Women with Urinary Incontinence: A Qualitative Descriptive Study

**DOI:** 10.3390/healthcare9030265

**Published:** 2021-03-02

**Authors:** Shelley Roberts, Zara Howard, Kelly A. Weir, Jennifer Nucifora, Nadine Baker, Leanne Smith, Heidi Townsend, Lynda Ross

**Affiliations:** 1 School of Allied Health Sciences, Griffith University, Gold Coast, QLD 4222, Australia; kelly.weir@griffith.edu.au; 2 Gold Coast Hospital and Health Service, Gold Coast, QLD 4215, Australia; Zara.Howard@health.qld.gov.au (Z.H.); Jennifer.Nucifora@health.qld.gov.au (J.N.); Nadine.Baker@health.qld.gov.au (N.B.); Leanne.Smith6@health.qld.gov.au (L.S.); hidsta@gmail.com (H.T.); 3 Menzies Health Institute Queensland, Griffith University, Gold Coast, QLD 4222, Australia; 4 School of Exercise and Nutrition Sciences, Queensland University of Technology, Brisbane, QLD 4059, Australia; l20.ross@qut.edu.au

**Keywords:** evidence-based practice, group interventions, peer support, pelvic floor muscle training, overweight/obesity, urinary incontinence

## Abstract

Urinary incontinence (UI) affects many women and impacts quality of life. Group-based interventions may be an effective and efficient method for providing UI care; however, interventions must be acceptable to patients to have an impact. This study aimed to explore patients’ perceptions of an exercise training and healthy eating group program (ATHENA) for overweight and obese women with UI. This qualitative descriptive study involved semi-structured interviews with a subset of participants sampled from a feasibility study of ATHENA. The ATHENA intervention was co-developed with end-users and implemented in Women’s Health Physiotherapy services at an Australian hospital. Interviews were recorded, transcribed and analysed thematically. Eleven female patients participated (mean ± SD age 54.2 ± 9.9 years; body mass index 30.5 ± 3.25 kg/m^2^). Participants found ATHENA highly acceptable, with three themes emerging from interviews: (1) Participants’ journey of change through ATHENA, describing the shifts in knowledge, attitudes, behaviours and symptoms participants experienced; (2) High satisfaction with ATHENA, including educational content, exercise components and delivery style; and (3) Group setting integral to ATHENA’s success, with participants providing support, building friendships, and facilitating each other’s learning. Overall, ATHENA was acceptable to participants, who provided each other with peer support; an unexpected moderator to ATHENA’s success.

## 1. Introduction

Urinary incontinence (UI) affects between a quarter to a half of adult women globally [1] and results in a significant personal and economic burden [2,3]. UI affects women’s daily activities and social roles; has negative impacts on intimacy and sexual function; and results in social isolation, with many women suffering alone [4,5]. It is also associated with significant shame and embarrassment; one Austrian study reported that UI is considered significantly more taboo than depression or cancer [6]. This shame impacts women’s quality of life and often prevents them from seeking treatment [4,7,8]. In fact, up to three-quarters of women with UI do not seek medical help for UI due to fear of embarrassment [7,8,9], and their attitudes towards UI treatment are often negative [4,5]. For those who do seek help, the average time between the onset of UI symptoms and reporting these to a health care professional (HCP) is over 13 years [10]. Many patients report feeling that UI is treated as a secondary problem or a nuisance by general practitioners and that many HCPs lack knowledge on, and/or interest in, UI [11]. Once a woman receives a referral for specialist medical care for UI in the Australian public health system, they often report experiencing poor transitional care between hospital and community settings and long wait times [11].

The pathophysiology of UI is often multifactorial and includes pelvic floor muscle dysfunction, obstetric injury and lifestyle factors, such as obesity [12], which carries its own stigma [13]. Evidence-based clinical practice guidelines recommend supervised pelvic floor muscle training (PFMT) as first-line treatment for women with UI, and weight loss for obese women with UI [14]. Physiotherapists with further training in continence and women’s health are well placed to provide such care, with input from other disciplines (such as dietitians to assist with weight loss). Recent studies on service models using primary contact advanced physiotherapists show improved access to care and reductions in wait times in Australian public health services [15,16]. Despite this, UI prevalence continues to rise with the ageing population and high burden of overweight and obesity; hence, innovations are needed to improve access to care that is both efficient and in line with evidence-based guideline recommendations. A recent randomised control trial has shown that a group-based approach to UI treatment is equally effective as standard, resource-intensive individual care, with participants experiencing a 74% reduction in the frequency of leakage at one-year post-intervention [17]. Group-based interventions are considered to optimise effectiveness for behavioural modification through peer support, mutual self-help, and increased motivation/compliance with treatment [18], and are likely to maximise delivery efficiency/accessibility in already stretched health services.

Our team has developed an innovative model of care to translate evidence-based guidelines for UI treatment into a “real world” setting for obese and overweight women. An exercise training and healthy eating group program for overweight/obese women with UI (ATHENA) was co-developed by a multidisciplinary team of academics, HCPs and a patient representative, using an integrated knowledge translation (i.e., co-production) approach [19,20]. It was designed to be delivered pragmatically in usual clinical practice to increase patient access to evidence-based UI care without placing a large burden on health services. Given the nature of UI as a condition, including the associated shame, and its impact on women’s health and daily activities, it is paramount to gain participants’ perceptions around the acceptability of a group-based UI intervention. The United Kingdom Medical Research Council guidance strongly recommends evaluating participant responses to complex interventions to understand why they do or do not work; gain insights into mediating factors and/or unexpected outcomes; and inform future implementation processes, to maximise the sustainability and cost-effectiveness of health services [21,22]. This study aimed to explore patients’ perceptions of and experiences with the ATHENA intervention for UI management, focusing on acceptability.

## 2. Materials and Methods

### 2.1. Study Overview

This nested, qualitative descriptive study involved semi-structured interviews with a subset of participants from a larger feasibility study of ATHENA [23]. The larger study involved developing, implementing and evaluating the ATHENA intervention within usual practice in a Women’s Health Physiotherapy clinic at an Australian hospital. ATHENA’s development involved translating evidence-based clinical practice guidelines for UI management into a 12-week program, as described in detail elsewhere [20]. An implementation-effectiveness hybrid type 3 study was conducted to concurrently assess: (a) implementation (via process evaluation, guided by the UK’s Medical Research Council guidance) and (b) effectiveness (via pre/post outcome assessment of UI symptoms and quality of life). Part of the predefined process evaluation included qualitative evaluation of participants’ responses to the intervention, via interviews. Due to the large volume of qualitative data, interview findings are reported here, while all other findings relating to ATHENA’s implementation and effectiveness are reported elsewhere [23]. The study received ethical approval through relevant hospital and university human research ethics committees.

### 2.2. Study Setting and Participants

The study was conducted within the Women’s Health Physiotherapy service at a large, tertiary public teaching hospital in Queensland, Australia. The service routinely provided individual, face-to-face appointments for women with UI from a urogynaecology waitlist. Participants included a subset of patients from a larger study, who had been enrolled in and completed the ATHENA program (described below). Eligibility criteria for the larger study included: female patients within the hospital’s Women’s Health Physiotherapy service, aged ≥18 years, with body mass index (BMI) of ≥25 kg/m^2^ and diagnosis inclusive of UI. Exclusion criteria included exercise contra-indications (acute illness, uncontrolled cardiovascular conditions), pregnancy, <3 months post-natal, and being unable to provide informed consent (poor cognition or non-English speaking background). Participants who had completed the ATHENA program were purposively sampled [24] from the larger study cohort to provide variation in age, weight and clinical condition. These patients were asked verbally (face-to-face or via telephone) if they would like to provide feedback in an interview. Those agreeing provided verbal consent (in addition to written informed consent for the larger study) to be interviewed. There was no pre-determined sample size; rather, participants were recruited until data saturation was reached [25].

### 2.3. Intervention

The ATHENA intervention [20] was co-developed by a team of multidisciplinary researchers and HCPs (women’s health physiotherapists, dietitians and allied health research fellows) and a health consumer researcher. A detailed description of the intervention and its development is published separately [20], but briefly, it involved: weekly, supervised PFMT; general exercise training; pelvic health education topics; and healthy eating education topics, adapted from the Healthy Eating and Lifestyle Behaviour-Change Program [26]. Each participant attended one session per week, for 12 consecutive weeks (with minimum attendance set at four weeks to be included in the study). All sessions included 10–20 min of PFMT and 30–40 min of general exercise training, supervised by physiotherapists in the outpatient gym. In addition, the first four sessions also included a 20–25-min pelvic health education and a 40–60-min healthy eating education. Both education components consisted of four topics each, which were rolling in nature, so patients had multiple chances to attend (e.g., if they missed a week), or re-attend (e.g., if they wanted a refresher). The pelvic health education included the following topics: Powerful Pelvis (definition, prevalence, risk factors and management of stress urinary incontinence); Beautifully Behaved Bladder (prevalence, risk factors and management of an overactive bladder); Terrific Number Twos (bowel function, stool types, bowel management); and Learning to Link (relationships between all three previous topics). The healthy eating education included: Enjoy Eating (Australian Guide to Healthy Eating, food groups, serve sizes); Powerful Portions (meal planning, portion control); Shop ‘til you Drop (reading nutrition labels, supermarket shopping tips); and Mood and Food (emotional eating, mindfulness, hunger/satiety cues).

A typical patient flow through the program included 2-h sessions for the first four weeks (with PFMT, general exercise training and pelvic health and healthy eating education sessions) and 1-h sessions for the remaining weeks (PFMT and general exercise training alone, once all education topics were completed).

### 2.4. Study Procedure

A semi-structured interview guide was developed consisting of questions relating to ATHENA, within four domains: (1) Overall experience; (2) Understanding components; (3) Participating in and using the program; and (4) Perceived value (see Supplementary File 1), and was piloted with HCPs and our consumer representative. Interviews took place after patients had completed the ATHENA program. Interviews were conducted via telephone (*n* = 7) or in person (*n* = 4), as per patient preference, by three members of the study team with experience in semi-structured interview techniques: one experienced Women’s Health Physiotherapist (ZH; clinician researcher) and two PhD-qualified dietitians (SR and LR; both academics/researchers). All interviewers were female and were involved in ATHENA delivery (ZH led around half of PFMT, general exercise and pelvic health education sessions; SR/LR each delivered around a quarter of healthy eating education sessions). Hence, there was a chance that interviewers had met participants previously; however, participants encountered many HCPs during ATHENA. Participants were aware that ATHENA was a new program being evaluated in a research study (as they had provided informed consent to the larger study) and were encouraged to be as open and honest as possible in their responses. A conversational style of interviewing was used to allow patients to tell a story and elicit detailed and relevant information. This involved active listening, using patients’ responses to guide the direction of the conversation and determine which prompts and questions to use next. The first question in each domain was broad and prompts were used as necessary to gain patients’ perspectives. After this question, interviewers asked patients about specific elements within that domain. Finally, each domain contained a checklist for interviewers to consult before moving onto the next domain. Interviews were recorded using a handheld, digital recording device and transcribed verbatim by an external transcription agency for later analysis by the research team. No repeat interviews were required. Interviewers took brief field notes after each interview.

### 2.5. Data Analysis

Interview data were analysed thematically using Braun and Clarke’s six-step guide to thematic qualitative analysis [27]. Transcripts were uploaded to a qualitative analysis computer software package (NVivo, version 11, QSR International Pty Ltd., Doncaster, Australia) and the following steps were undertaken:

1. Data familiarisation: Two authors (ZH and SR) read and reread transcripts to become immersed in the data.
2. Generating initial codes: One author (ZH) highlighted key quotes and initial codes were developed based on participants’ verbatim statements.
3. Searching for themes: Using NVivo, ZH started to group codes according to similarity, into potential themes and/or subthemes, ensuring all data were represented.
4. Reviewing potential themes: At this stage, another author (SR) reviewed all grouped codes to consider their relevance and coherence to that potential theme/subtheme. This resulted in the reorganisation of some codes, such as merging groups of codes together or moving certain codes to another group. This was an iterative process involving frequent discussions between ZH and SR, and concept mapping of emerging themes/subthemes. Authors regularly referred back to original transcripts to ensure groupings accurately represented the data and no data were missed. After several rounds of iterations, subthemes were grouped into coherent and distinctive themes.
5. Defining and naming themes: SR and ZH continued to revise and refine the descriptions and labels for each theme/subtheme until all data were adequately represented, and the overall “story” of the data was clear. These continued to be revised iteratively with other authors’ input.
6. Producing a report: Writing up of findings involved presenting the data in a detailed and logical way, with compelling participant quotes supporting each subtheme.

Steps were taken to enhance the trustworthiness of findings [28], including: training interviewers and using a standardised, semi-structured interview guide (for dependability); giving detailed descriptions of study context, the selection and characteristics of participants, and data collection and analysis processes (for transferability); and using purposive sampling for richer variation in perspectives, ensuring all relevant data were represented and including participant quotes in our findings (to optimise credibility). Transcripts were not returned to individual participants for member checking; however, the consumer researcher (patient representative) assisted with data interpretation where appropriate.

## 3. Results

### 3.1. Demographics

Eleven female patients participated in interviews, which ranged from 10–30 min in length. Their characteristics are outlined in Table 1. All participants had a primary diagnosis of UI (including stress, urge, or mixed), with most reporting a long history and high severity of UI symptom bother. Participants’ primary goals were to improve continence, reduce weight and strengthen PFMs by the program’s end. Some patients also outlined relationship and community participation goals they were hoping to achieve with improved continence. Of the patients invited to participate in an interview, nil declined, but two were unwell at their scheduled interview time and could not be rescheduled within the study time frame.

### 3.2. Interview Findings

**Figure 1:**
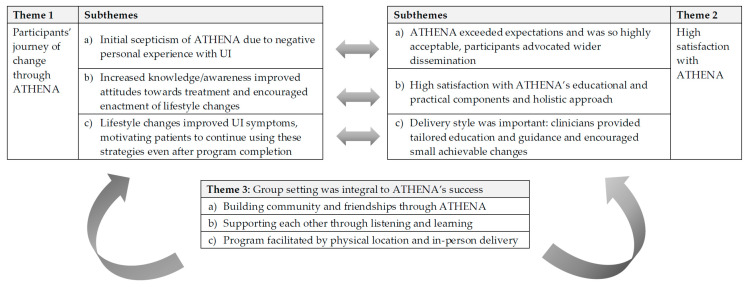
Conceptual model of ATHENA’s mechanisms of action. Note: Themes 1 and 2 interacted with each other, in that: (i) despite initial scepticism of ATHENA (due to negative personal experience with UI), participants’ expectations were exceeded and they found the program highly acceptable; and (ii) educational and practical components of ATHENA (and the way in which they were delivered) were found to be highly acceptable, and resulted in increased knowledge/awareness about UI, improved attitudes towards treatment, enactment of lifestyle changes and, consequently, improvement in UI symptoms. Theme 3 was overarching and acted as a moderator to the intervention as a whole.

Overall, participants expressed positive perceptions of the ATHENA program and were highly satisfied with it. Their responses formed three themes, each with three subthemes, described in Figure 1 and in detail below.

#### 3.2.1. Theme 1. Participants’ Journey of Change Throughout the ATHENA Program

This theme describes the changes participants experienced throughout their ATHENA journey, including shifts in their attitudes and behaviours, and physical changes in their UI symptoms. Each subtheme below represents the steps patients took on this journey of change, illustrating how patients perceived ATHENA to work.

(a) Initial scepticism of ATHENA due to negative personal experience with UI

Many participants were doubtful that any treatment could improve their symptoms, including ATHENA. Several women said that prior to attending, they were sceptical of whether it would work or were uncertain of what it would involve. Others described being hesitant, nervous or embarrassed, or having low motivation to attend.

*“Will it work? Really work? You’ve got this suspicion.”*

*P03*

*“I was hesitant at first, given the nature of what we’re doing there, [and it] being very personal.”*

*P02*

Participants went on to describe their experiences of living with UI and how it negatively impacted their lives. Most expressed feeling ashamed and embarrassed, and some previously avoided talking about or seeking help for their UI due to this shame. Some patients explained how they had come into the program only after they had the courage to *“open up”* to their doctor about their UI. Some expressed the belief that UI only affected *“old people”*. Many participants explained how, prior to attending ATHENA, they had little hope for improvement as they had suffered with UI for many years with no relief. Some patients explained how they had given up or resigned themselves to living with UI for the rest of their lives.

*“I’ve had incontinence for a long, long, long, long time. It affected…my everyday life to the point where I was wearing disposables… I had resigned myself to a life of using pads and not being able to run… [I was] embarrassed because…the leak’s been so, so, so severe that it has come through my clothes.”*

*P07*

*“I lived with my condition thinking, because I’m old, I would have to just always not drink when I go out, and just watch out, and put pads on, because I’m old.”*

*P11*

(b) Increased knowledge/awareness improved attitudes towards treatment and encouraged the enactment of lifestyle changes

Participants described how much they learned from ATHENA and appreciated the knowledge and awareness they gained around UI and their own bodies.

*“There was just so much that I didn’t know, or I hadn’t thought of, and it was actually putting it all into perspective…. just being aware of things… because you try not to think about it, because you’re ashamed… being self-aware was really, really good. That was probably the biggest change for me, that self-awareness.”*

*P02*

This learning led to significant changes in participants’ attitudes towards UI treatment, giving them renewed optimism that their condition could be improved. For example, many women expressed surprise upon learning that training their PFMs could greatly reduce UI symptoms. Several expressed quite stark changes in their mindsets towards UI and felt more positive about the future:

*“It changed me in the way that I think… it challenged me to act in a good way.”*

*P02*

*“What’s amazing [is], I learned that you could, after 60—I’m turning 65 this year —I learned that I could still train my muscle.” *

*P11*

With positive shifts in knowledge and attitudes towards UI and its treatment, patients felt more optimistic and motivated to enact the lifestyle strategies they had learned during the program. They felt confident to perform PFMT at home, after being shown by physiotherapists and practicing in the ATHENA gym, and felt supported and encouraged to achieve their goals.

*“You’re aware of the changes. You’ve [been shown] how to do it, but then just seeing how we’re actually implementing it… how you can make adjustments.” *

*P02*

*“Now when I get up, before I get up, I do my pelvic [floor] exercise... I do them when I do the dishes. I do them when I hang up the washing. I do them in the car when I’m in traffic. I do them at every opportunity.” *

*P11*

(c) Lifestyle changes improved UI symptoms, motivating patients to continue using these strategies even after program completion.

After attending several sessions, participants started to notice positive and meaningful changes in their UI symptoms. This further encouraged and motivated them to continue doing exercises and implementing lifestyle changes at home. Patients described several improvements in their physical and mental health, some of which were quite stark:

*“You know, this is amazing for me, because we didn’t go out anymore. I never wanted to do anything. I was always scared I would have problems with incontinence. I don’t have that anymore. It’s a new life.” *

*P11*

*“If you asked me six months ago that the changes that have occurred now, I wouldn’t have believed you that they were possible because I’d put up with it for so long the way it was.” *

*P02*

Many participants said learnings during the program helped them to take responsibility for their own health, and they planned to continue with the changes they had implemented even after the program finished. A few women were worried about maintaining these behaviour changes, as the program had provided great motivation for them. However, many were adamant that they would not regress after seeing such significant improvements in their UI; as one said, *“I’m not going backwards” (P06)*.

*“It doesn’t have to stop here…I still do [pelvic floor] exercises when I…take my shower in the morning, when I wake up, in the bed, and then when I drive, I try to do some movement. Everywhere I go now, I’m conscious.” *

*P03*

#### 3.2.2. Theme 2. High Satisfaction with ATHENA

This theme outlines participants’ very high satisfaction with the ATHENA program overall. The program exceeded their initial expectations so much that they became advocates for the program and wanted it to be widely available to other women. Participants spoke about the value of each topic/component and how it all linked together to provide a holistic approach to UI treatment. Participants liked the delivery style that physiotherapists and dietitians adopted in ATHENA, which was described as individualised and flexible, rather than generic or rigidly prescriptive.

(a) ATHENA exceeded expectations and was so highly acceptable, participants advocated wider dissemination

Despite having low initial expectations and doubting ATHENA would help with their UI, all participants were highly satisfied and explained how *“it was better than I thought it was going to be” (P19)*. Patients said they enjoyed the program and found it fun, interesting, informative and beneficial. Their initial fears of attending were unfounded, as one participant described what surprised her most: *“It was actually how comfortable I felt, to be honest!” (P02)*.

*“It was absolutely perfect for me in my situation.” *

*P07*

*“I didn’t have any expectations, though I was hoping it would help me improve my pelvic floor, obviously, and it did.” *

*P09*

Participants were so satisfied with ATHENA that they desired its wider dissemination so others could benefit as they did. All participants said they would recommend the ATHENA program to others, as one said, *“I already actually have—a lady at work. She wants to know all about it” (P05)*, and several actively encouraged the research team to enrol more patients or advertise more broadly. There was a sense of urgency from some participants, emphasising that “people don’t know” about the program. One patient thought testimonials could promote the program and help women decide on whether to enrol, given the uncertainty she faced prior to attending.

*“I think more awareness [of ATHENA is needed]… just send email and a brochure or something… I think we need to target more people.” *

*P03*

*“I’ve never done any other program like this. I think lots of women would benefit if they knew about, but they don’t.” *

*P11*

(b) High satisfaction with ATHENA’s educational and practical components and holistic approach

Participants were very satisfied with each component of ATHENA, including the educational content and hands-on/practical activities. Some women placed higher importance on certain topics that were relevant to them, but expressed all were beneficial. They especially liked how all the components linked to give a holistic approach to UI treatment. The educational content was new to some, whilst others said it was a good “refresher” of what they already knew.

*“I think the combination of the exercise, with emphasis on the pelvic floor exercise, and the dietitian—it was perfect.” *

*P11*

*“Well, to me, it was actually understanding how the pelvic floor all linked in together. I just didn’t even think about that before.” *

*P02*

All participants found the exercise components (supervised PFMT and general exercise, guided by physiotherapists) very helpful, as it helped them to not only strengthen their PFMs, but also to be more mindful of their pelvic floor in daily life. Being aware of how to properly engage their pelvic floor was important to participants, and physiotherapists helped them do this.

*“Well, all the exercises we did were really beneficial for us… those exercises helped me to be more mindful of switching on [pelvic floor muscles] when doing particular movements.” *

*P07*

*“It made me think again about my pelvic floor more, whereas sometimes you squeeze everything in, your bottom and everything, whereas now when I’m doing my exercises I know exactly where I’ve got to squeeze.” *

*P09*

Most participants found the nutrition education (led by dietitians) to be very helpful:

*“Probably the nutrition [topic helped me most] …there was a few little tips in that for me… I can see there is extra changes that I can probably do, and have been… I’m more aware of what I’m doing and when I’m snacking and…emotional eating. Because it becomes a habit…and it’s trying to break that cycle.” *

*P19*

Surprisingly, several participants found the bowel education was most useful to them:

*“I didn’t understand the pelvic floor muscle is also supporting your bowels… I really, really got a lot out of that particular part… when she spoke about constipation—I suffered from that quite a lot and really understanding that I thought, oh wow… it really helps. It really does help.” *

*P07*

(c) Delivery style was important: clinicians provided tailored education and guidance and encouraged small achievable changes

From participants’ responses, it was evident that the delivery method of each component was just as important as the content itself. They liked that the information was easy to understand and tailored to individuals:

*“All of this information, I’m able to apply it to me… for my circumstances. That was probably the most beneficial; that it was all applicable to me, rather than trying to sift through information I’d found myself. It was relative and easy enough to understand.” *

*P02*

Patients also liked the HCPs who delivered ATHENA, describing them as friendly and approachable, and said they made patients feel comfortable and confident to participate/ask questions. Participants also valued HCPs taking the time to explain topics in different ways and ensure all participants understood the content; as one said: *“You spent time with each of us when we needed it” (P06).*

*“The girls were quite good [at] going through everything and making sure that you understood everything. Even drawing diagrams to illustrate when you didn’t, which was helpful... There was always plenty of time for questions and… we were given the information pamphlets if we wanted to take them away.” *

*P09*

Participants placed high trust in HCPs’ knowledge and skills and noticed teamwork amongst them, even when locums replaced regular staff who were away. While perceptions of HCPs were overwhelmingly positive, one patient thought locum HCPs could remind patients more often about holding their PFMs during the exercise component.

*“Your skill, you were on the same level… Even if you’re not here, someone else said the same thing… I got this connection, ‘ah, okay, this is really a team.’ You work like a team and I was happy that all your physiotherapy was on the same line. You get the same thing with everybody.” *

*P03*

Participants liked the fact that HCPs provided guidance rather than being rigidly prescriptive, and had an encouraging and personal approach.

*“[Initially] I was saying, ‘if you’re going to try to change my diet, I’m not coming’… I know my diet’s bad. People for years have been trying to change me… [in ATHENA] they’re not telling you to change. You’ve just given us the tools... I’m 49 years old next month and I don’t want to be lectured about my diet, and I found it wasn’t a lecture, which is good. It was just knowledge, which was good.” *

*P19*

Participants valued HCPs focusing on making small, achievable changes that were meaningful to them personally:

*“Whether it was the diet or the exercise… there’s just so many small changes that have been made, and I think that was the best; that they were small…changes…and it was very obtainable.” *

*P02*

#### 3.2.3. Theme 3. Group Setting Was Integral to ATHENA’s Success

Participants frequently mentioned the group setting was one of the most important aspects of the program. They spoke about having support from other women and feeling safe, comfortable and confident to discuss UI. They found solace in knowing that others were going through the same issues. Surprisingly, many found friendships with the other participants and said they had fun, laughed and shared stories. Many thought the group setting motivated them to attend and made it easier to participate.

(a) Building community and friendships through ATHENA

Participants described how the group environment allowed them to connect with other women experiencing UI. Several explained how it was comforting to know others had been through similar issues, and took solace in knowing they were no longer alone:

*“It’s good to know that there’s other women with the same problem. You’re not on your own.” *

*P08*

*“I liked the people that were there, so I was very motivated, because other people were in the same situation as I was.” *

*P11*

Participants described how attendees varied in terms of age, background, culture and severity of UI. This was described as a benefit rather than a barrier, with many women suggesting their differences brought them closer together and helped them feel included due to a common goal:

*“Everybody’s at totally different levels of sickness, and ages and things like that, so it made it more acceptable to be here... it felt a lot easier to be part of the group.” *

*P02*

*“Just the variety of everybody’s different problems. It’s the same problem but yet, it’s so different for each person. I thought you’re either weak or you’re not, so just learning about those different areas, I think.” *

*P19*

Many participants described building new friendships during the program. Several women said these friendships motivated them to keep attending ATHENA or to participate more actively in the exercise training sessions. Some participants said they became very close as a group, which made the program more enjoyable overall.

*“It’s easier because you’ve got those friendships happening and… you want to come [to sessions]. You weren’t there by yourself being stressed out or pushed too hard. The others helped you do it.” *

*P06*

*“You meet new people. You’re sharing life with other women that have gone through the experience, which makes it easier. Yeah, and you create social friendships.” *

*P01*

(b) Supporting each other through listening and learning

Many participants discussed how valuable the group environment was for providing support and assistance to each other. To many women, this was considered as important as the treatment itself, as they had felt alone and unsupported for so long in their struggles with UI. Several women explained how helpful it was to talk and listen to each other, as they learned from each other’s experiences and provided suggestions to others.

*“As a group it worked as well, because we all had a bit of input, or output listening to other people’s problems, situations. It was good… Everyone else seems to listen to what you said, as much as you listening to someone else… You listen and take a bit of their problem and you can relate it to yours, to get answers as well.” *

*P06*

*“We’re all in the same situation. We’re talking about what’s causing, what’s going on at home, the stress of different things…to be able to talk about life—it’s like a nice counselling situation.” *

*P01*

Participants expressed there was a sense of collegiality amongst them. They felt that they were “all in this together” and bonded over stories and experiences of living with UI. Many said they were comfortable sharing things that they had previously been too ashamed to tell anyone, and felt relief from being able to finally speak about it:

*“Given the nature of what we were discussing… it’s not something you like to be open with. But it felt like a really safe, inclusive environment, very supportive rather than… being judged... so, that was really, really good for me.” *

*P02*

Some women said that they learned more during the program due to other participants asking questions, and through group discussions. A few women described how they made an effort to look out for other participants by helping them learn or perform their exercises properly.

*“I think the group setting is the best…just for the fact that some of the other women would ask questions and I thought ‘oh, I never thought about that. I want to know about that too.’ So just for that fact as well.” *

*P07*

Several participants expressed that connecting with other women of varying ages, backgrounds and UI severity, and listening to their stories and experiences, helped them to understand more about their condition or helped put their own issues into perspective.

*“Different ages… like 30 into their 60 s. I suppose I didn’t realise how widespread the condition is.” *

*P08*

*“I talked to the other women with similar sort of problems. Then I found out a lot of people were worse off than I am! So, I felt better about that, not for them, but for myself… I know it’s a bit selfish, but...” *

*P10*

(c) Program facilitated by physical location and in-person delivery

The final aspect of the group setting that participants spoke about was the physical location of sessions. Participants liked attending in person and thought this was essential as they could talk with other participants, ask questions of staff and practice their exercises under the supervision of a physiotherapist.

*“[Attending in person] helped the program to be successful, because women and men come together as tribes and share. That’s just human instincts… you won’t get as much of an outcome if you’re going to do it by Skype or…through social media or something. You need the one-on-one. You need the group input.” *

*P01*

All participants said the location of the program (at the hospital) was convenient and relatively easy for them to access. Some took public transport while others travelled by car; however, parking availability and cost were challenges for some. Work was a barrier to attendance for some participants, as it was difficult to obtain time off. A few participants expressed a desire to continue meeting up as a group at an alternative location, even after the program finished:

*“If it was outdoor, you know…like a centre that is made for women and gathering all the women with this situation and we can…continue it.” *

*P03*

Participants appreciated the hospital setting in which the program was conducted and said the weekly, in-person attendance provided protected time for them to perform their exercises, as many found it difficult to find time previously. Some said that knowing other participants would be there provided extra motivation to attend, and others finally felt that they could prioritise their health over other demands:

*“You don’t find time for you. When you get time, you’re always think about something else; your family, your parents… [with] this, I knew I was scheduled for an appointment here, I made it…because I have to make it; I know you’re waiting for me.” *

*P03*

## 4. Discussion

This study explored participants’ perceptions of and experiences with an exercise training and healthy eating group lifestyle program (ATHENA) for overweight and obese women with UI. Participants found ATHENA highly acceptable, valuing the intervention components and how it all came together in a holistic way. The educational content and its delivery by HCPs were seen to positively influence participants’ knowledge, attitudes, behaviours and outcomes. The group setting was pivotal to success, acting as a moderator to intervention effectiveness through the building of community, friendships and support networks among participants. Participants were passionate about ATHENA, given their positive experiences with it, and wished to make it available to all women suffering with UI.

Overall, participants found ATHENA highly acceptable, despite having initial scepticism of its potential effect or hesitancy around attending a group program due to shame or embarrassment. Previous research confirms our findings that women’s personal experiences of living with UI and the shame associated with it negatively affected their lives and perceptions of treatment [4,5,8,9]. Yet, this study demonstrated substantial shifts in participants’ knowledge, attitudes and beliefs around UI after attending ATHENA, despite initial negative perceptions. These shifts changed participants’ outlooks towards UI and motivated them to implement lifestyle changes they had previously dismissed as being futile. As a result, many women experienced improvements in their UI symptoms, reinforcing continued behaviour change. The effects were so substantial for some, they described having “a new life”. This “journey of change” through ATHENA appeared to explain, at least in part, the mechanism of action behind the intervention.

Importantly, the group environment appeared to be a moderator of ATHENA’s effectiveness, with participants describing this as one of the most important aspects of the intervention. The friendships participants built provided them with motivation to attend and engage in ATHENA sessions, and many said other participants facilitated their learning by asking questions of staff or sharing their experiences. This is congruent with Bandura’s Social Cognitive Theory, which states that learning occurs in a social context, with dynamic and reciprocal interactions between an individual (with their own set of learned experiences), environment (external social context) and their behaviour [30]. This concept, known as “reciprocal determinism”, is illustrated in the current study by ATHENA participants acknowledging how their own personal experiences with UI negatively affected their attitudes towards treatment; how this changed upon attending ATHENA (in a social environment alongside other women with similar issues); and how this subsequently motivated them to enact behaviour and lifestyle changes.

Other Social Cognitive Theory constructs were apparent in this study; for example, participants expressed engaging in “observational learning” by watching and reproducing the actions of others (e.g., physiotherapists or other participants in the gym) or being motivated by other participants’ successes. “Reinforcement” of behaviour changes enacted by participants was evident in their perceived improvements of UI symptoms, despite having initially low “expectations” based on their past experience of UI. Finally, “self-efficacy” was increased through successes in enacting lifestyle changes and seeing results, which participants indicated was at least in part due to the way in which ATHENA was delivered. Staff provided tailored education and advice that was personally relevant; guidance and encouragement rather than prescriptive care; and focused on small, achievable and meaningful changes, which patients suggested contributed to positive attitude shifts and adherence with the program. The interactions between participants, the group environment and their behaviour appeared to be like a positive feedback loop, with participants feeding off each other’s positivity and successes.

Another benefit of the group setting was the peer support it provided. Participants expressed relief in feeling that they were no longer alone and no longer had to hide; they could talk about their UI openly with other women, which many had never done before. Research suggests that many women silently endure UI alone, as they feel too ashamed to talk about it or seek help [5], and importantly, women who experience greater social rejection, social isolation and internalised shame are more likely to have negative attitudes towards UI treatment [4]. This likely explains the substantial impact the group setting had on participants, as they had felt alone and isolated with their UI for so long. Some women were so satisfied with the program and the friendships they made, they wanted to continue meeting up and doing their exercises together, even after the program finished. Several participants also strongly desired ATHENA to be available to other women experiencing UI, and encouraged staff to advertise it widely.

This study has some limitations. First, it is a small single-site study of eleven women, whose perceptions may not be representative of all women with UI. However, purposive sampling was used for maximum variation in participants and interviews were continued until data saturation was reached. Second, interviewers were involved in delivering some ATHENA sessions, so participants may not have felt comfortable expressing negative perspectives of the program. To minimise this, most interviews were conducted over the phone and at least a month after program completion (and participants were exposed to many different staff during their ATHENA sessions). Additionally, as several participants provided constructive feedback, it is likely that these participants felt comfortable providing honest responses.

## 5. Conclusions

This study found that a group-based exercise training and healthy eating program (ATHENA) was highly acceptable to women with UI and had many perceived benefits for participants. The group setting, initially perceived to be shameful or embarrassing to attend, amplified the effects of the intervention and resulted in unexpected outcomes such as friendships and peer learning and support. The group approach appeared to be a key factor in the program’s success, with participants expressing that it increased their motivation to attend and to implement lifestyle changes at home. This study also showed that women with UI, who had previously given up hope of recovery and who were sceptical of a group program for UI, could experience a transformative change in their knowledge, attitudes and behaviours, ultimately resulting in improvements in their UI symptoms and quality of life. Participants’ high acceptability of the ATHENA program suggests that with the right approach, the seemingly impossible can be achieved. Health care organisations looking to implement their own evidence-based UI interventions into usual care should consider group-based approaches, for the reasons outlined in this paper, and for efficient health care delivery.

## Figures and Tables

**Table 1:** Participant characteristics.

Characteristic	Mean (±SD)	Range
Age (years)	54.2 (±9.9)	31–77
BMI (kg/m^2^)	30.5 (±3.25)	25.2–45.9
UI severity ^a^		
Bladder dysfunction	4.7 (±1.5)	2.4–7.1
Bladder bother	2.6 (±0.7)	1–3
Bowel dysfunction	1.8 (±1.0)	0.3–3.4
Bowel bother	0.9 (±0.8)	0–2

BMI: Body mass index, UI: Urinary incontinence, ^a^ UI severity determined at baseline using the Australian Pelvic Floor Questionnaire [29]. Bladder and bowel dysfunction scores range from 0–10 and bladder bother scores range from 0–3, with higher scores indicating higher dysfunction and bother.

## Data Availability

The data presented in this study are available on request from the corresponding author. The data are not publicly available as this was not approved in the ethics application.

## Supplementary Materials

The following are available online at https://www.mdpi.com/2227-9032/9/3/265/s1, Supplementary File 1: Interview guide.

## References

[1] Milsom I., Gyhagen M. (2019). The prevalence of urinary incontinence. Climacteric.

[2] Coyne K.S., Wein A., Nicholson S., Kvasz M., Chen C.-I., Milsom I. (2013). Comorbidities and personal burden of urgency urinary incontinence: A systematic review. Int. J. Clin. Pract..

[3] Milsom I., Coyne K.S., Nicholson S., Kvasz M., Chen C.-I., Wein A.J. (2014). Global Prevalence and Economic Burden of Urgency Urinary Incontinence: A Systematic Review. Eur. Urol..

[4] Wang C., Li J., Wan X., Wang X., Kane R.L., Wang K. (2015). Effects of stigma on Chinese women’s attitudes towards seeking treatment for urinary incontinence. J. Clin. Nurs..

[5] Mendes A., Hoga L., Gonçalves B., Silva P., Pereira P. (2017). Adult women’s experiences of urinary incontinence: A systematic review of qualitative evidence. JBI Database Syst. Rev. Implement. Rep..

[6] Elenskaia K., Haidvogel K., Heidinger C., Doerfler D., Umek W., Hanzal E. (2011). The greatest taboo: Urinary incontinence as a source of shame and embarrassment. Wien. Klin. Wochenschr..

[7] Larsson G., Leppert J., Hägglund D., Walker-Engström M.-L. (2003). Reasons why women with long-term urinary incontinence do not seek professional help: A cross-sectional population-based cohort study. Int. Urogynecol. J..

[8] Margalith I., Gillon G., Gordon D. (2004). Urinary incontinence in women under 65: Quality of life, stress related to incontinence and patterns of seeking health care. Qual. Life Res..

[9] Hägglund D., Wadensten B. (2007). Fear of humiliation inhibits women’s care-seeking behaviour for long-term urinary incontinence. Scand. J. Caring Sci..

[10] Grzybowska M.E., Wydra D., Smutek J. (2015). Analysis of the usage of continence pads and help-seeking behavior of women with stress urinary incontinence in Poland. BMC Women’s Health.

[11] St John W., James H., McKenzie S. (2002). “Oh, that’s a bit of a nuisance”: Community-dwelling clients’ perspectives of urinary continence health service provision. J. Wound Ostomy Cont. Nurs..

[12] Wyndaele M., Hashim H. (2017). Pathophysiology of urinary incontinence. Surgery (Oxford).

[13] Puhl R.M., Heuer C.A. (2010). Obesity Stigma: Important Considerations for Public Health. Am. J. Public Health.

[14] National Institute for Health and Care Excellence (2019). Urinary Incontinence and Pelvic Organ Prolapse in Women: Management.

[15] Brennen R., Sherburn M., Rosamilia A. (2019). Development, implementation and evaluation of an advanced practice in continence and women’s health physiotherapy model of care. Aust. N. Z. J. Obstet. Gynaecol..

[16] Howard Z. (2018). Outcomes of a physiotherapy-led pelvic health clinic. Aust. N. Z. Cont. J..

[17] Dumoulin C., Morin M., Danieli C., Cacciari L., Mayrand M., Tousignant M., Abrahamowicz M. (2019). Group physiotherapy compared to individual physiotherapy to treat urinary incontinence in older women: A non-inferiority randomized control trial. International Continence Society 49th Annual Meeting.

[18] E Lamb S., Pepper J., Lall R., Jørstad-Stein E.C., Clark M.D., Hill L., Fereday-Smith J. (2009). Group treatments for sensitive health care problems: A randomised controlled trial of group versus individual physiotherapy sessions for female urinary incontinence. BMC Women’s Health.

[19] Canadian Institute of Health Research (2012). Guide to Knowledge Translation Planning at CIHR: Integrated and End-of-Grant Approaches.

[20] Howard Z., Ross L., Smith L., Baker N., Nucifora J., Townsend H., Weir K., Roberts S. (2020). An Exercise Training and Healthy Eating Group Program (ATHENA) for Overweight and Obese Women with Urinary Incontinence: An Intervention Description. Healthcare.

[21] Craig P., Dieppe P., MacIntyre S., Michie S., Nazareth I., Petticrew M. (2008). Developing and evaluating complex interventions: The new Medical Research Council guidance. BMJ.

[22] Moore G.F., Audrey S., Barker M., Bond L., Bonell C., Hardeman W., Moore L., O’Cathain A., Tinati T., Wight D. (2015). Process evaluation of complex interventions: Medical Research Council guidance. BMJ.

[23] Howard Z., Ross L., Weir K., Baker N., Smith L., Nucifora J., Townsend H., Roberts S. (2021). A group program for overweight and obese women with urinary incontinence (ATHENA): An implementation-effectiveness hybrid type 3 study. Int. Urogynecol. J..

[24] Palinkas L.A., Horwitz S.M., Green C.A., Wisdom J.P., Duan N., Hoagwood K. (2015). Purposeful Sampling for Qualitative Data Collection and Analysis in Mixed Method Implementation Research. Adm. Policy Ment. Health Ment. Health Serv. Res..

[25] Fusch P.I., Ness L.R. (2015). Are we there yet? Data saturation in qualitative research. Qual. Rep..

[26] Langston K., Ross L.J., HlthSc A.B.B., HlthSc R.H.B. (2020). Secondary-prevention behaviour-change strategy for high-risk patients: Benefits for all classes of body mass index. Nutr. Diet..

[27] Braun V., Clarke V., Cooper H. (2012). Thematic analysis. APA Handbook of Research Methods in Psychology: Vol. 2. Research Designs.

[28] Graneheim U., Lundman B. (2004). Qualitative content analysis in nursing research: Concepts, procedures and measures to achieve trustworthiness. Nurse Educ. Today.

[29] Baessler K., O’Neill S.M., Maher C.F., Battistutta D. (2010). A validated self-administered female pelvic floor questionnaire. Int. Urogynecol. J..

[30] Bandura A. (1986). Social Foundations of Thought and Action. Englewood Cliffs NJ.

