# Supplementary material for: Patient Perceptions of a Group-Based Lifestyle Intervention for Overweight Women with Urinary Incontinence: A Qualitative Descriptive Study
Source: Healthcare (Basel). 2021 Mar 2;9(3):265. doi: 10.3390/healthcare9030265 (PMC8001695; doi:10.3390/healthcare9030265)
Supplement: Supplementary file 1 [file healthcare-09-00265-s001.pdf]

Supplementary File 1: Interview guide

Semi-structured interview guide for patients
Preamble: "Thanks for taking the time to talk with me today. In this interview, I am going to ask you a few questions relating to your experiences with the ATHENA program. We want to hear your story so we can understand what patients think about this program. There are no right or wrong answers. Before we start, do you have any questions about the interview?"
**Domain 1: Overall experience with the intervention**
1. What did you think about this program overall?<br>2. What did you like / what didn't you like about the program?<br>3. Was there anything missing / anything you wish was covered in the program?<br>4. Could we have done anything differently?
**Domain 2: Remembering and understanding the intervention**
1. What were the main topics covered in the program?<br>(Prompts: those relating to diet / those relating to exercise)<br>2. Which topics were most useful to you / helped you the most?<br>3. Did you learn anything new?<br>4. Was there anything that surprised you about the program or what you learned?<br>5. Were there any topics you didn't understand?<br>6. Did the program meet your expectations?
**Domain 3: Using and participating in the intervention**
1. What did you think about the way the program was delivered (i.e. in person, group setting)?<br>2. Was it inconvenient to attend in person?<br>3. Did the group setting make it easier or harder to get involved / make lifestyle changes?<br>4. Did you do anything differently / change anything at home (i.e. eating or exercise routines) as a result of attending this program? If so, why? If not, why not?
**Domain 4: Perceived value of the intervention**
1. Was the program valuable to you personally?<br>2. Do you think the program worked? (i.e. did you lose weight or experience improvements in your incontinence?) If so, why? If not, why not?<br>3. Did you experience any other changes from attending this program?<br>4. Will you keep using what you have learned in the program, even after you finish it?<br>5. Would you recommend others to attend this program?
**Prompts:** If a question is answered with a simple yes or no, ask the patient to explain further. For example, you can ask "why" or "why not"?<br>Other generic prompts include: ""Can you tell me more about this?"; "Can you explain this further / expand on this?" and "What do you mean when you say ____?"
